# Overwintering and breeding patterns of monarch butterflies (*Danaus plexippus*) in coastal plain habitats of the southeastern USA

**DOI:** 10.1038/s41598-023-37225-7

**Published:** 2023-06-27

**Authors:** Michael R. Kendrick, John W. McCord

**Affiliations:** South Carolina Department of Natural Resources, Marine Resources Research Institute, 217 Fort Johnson Road, Charleston, SC 29422 USA

**Keywords:** Population dynamics, Conservation biology

## Abstract

Understanding variability in species’ traits can inform our understanding of their ecology and aid in the development of management and conservation strategies. Monarch butterflies (*Danaus plexippus*) are native to the western hemisphere and are well-known for their long-distance migrations but have experienced significant population declines in recent decades. Here we use a 5-year capture-mark-recapture dataset to compare monarch distributions, mating activity, and larval host plant use between two coastal plain habitats in South Carolina, USA. We observed seasonally specific habitat use, with maritime habitats serving as overwintering areas while nearby inland swamps support significant breeding in spring, summer, and fall seasons due to an abundance of aquatic milkweed (*Asclepias perennis*). We also observed mating activity by fall migrating monarchs and their use of swallow-wort (*Pattalias palustre*) in the spring as an important larval host plant in maritime habitats. This phenology and habitat use of monarchs diverges from established paradigms and suggest that a distinct population segment of monarchs may exist, with significance for understanding the conservation status of monarch butterflies and associated habitats in eastern North America. Further research should explore how monarchs along the Atlantic coast of North America relate to other eastern monarch populations.

## Introduction

Variability in the distribution and phenology of individual species across spatial and environmental gradients can highlight how ecological factors allow realized niches to be manifested from their broad theoretical ones^1,2^. Range-wide variability in species’ traits and associated environmental conditions, both of which help to determine realized niches^3^, can be important for the appropriate parameterization of trait-based distribution models^4,5^ and has important implications for understanding ecological interactions^6,7^. For widely dispersed species, understanding trait variability is particularly important since populations may interact with their environment in different ways across their geographic range. Sensitivity to overwintering conditions, for instance, is known to vary across invertebrate populations^8^.

Monarch butterflies (*Danaus plexippus*, hereafter monarchs) are found across much of the globe inhabiting North and South America, the Indo-Pacific islands, Australia, New Zealand, and parts of southern Europe^9^. This widespread distribution has led to a diversity of morphologies^10,11^, life histories^12^, and population genetics^13,14^. Monarch traits have been documented throughout much of the species’ native range in North and South America, including for the eastern monarch population^15^ situated east of the Rocky Mountains, the western monarch population^16^ situated west of the Rocky Mountains, as well as for South American populations^11^. The eastern monarch population is often defined by sharing several key traits which include reproductive diapause during its fall migration to overwintering grounds in Mexico^17^ and heavy reliance on, and migration synchrony with, the phenology of several milkweed species. This includes the common milkweed, *Asclepias syriaca*, swamp milkweed, *A. incarnata*, and green milkweed, *A. viridis*^18^. Not all monarch populations share these key traits^11^, however, with fall monarchs in the southeastern US showing evidence of reproductive activity^19^ and heavy reliance on other species of *Asclepias* as well as other genera within the family Apocynaceae that are native to the local environments^20,21^. While distinguishing populations of monarchs can be challenging^18^, variability in these traits, and the fitness associated with them, may aid in distinguishing monarch populations from one another^11^.

This study documents key behavioral and life history traits associated with monarch butterflies in two coastal plain habitats of coastal South Carolina (hereafter SC) in the southeastern United States. Its major findings include documenting the phenology of monarch habitat use by comparing the distribution, breeding activity, and plant associations across maritime and inland swamp habitats. By contrasting these traits from monarchs in coastal plain environments with more well-studied central US and western monarch populations, resource managers can better develop conservation and natural resource management plans for monarch butterflies.

## Results

### Seasonal distribution patterns

**Figure 1:**
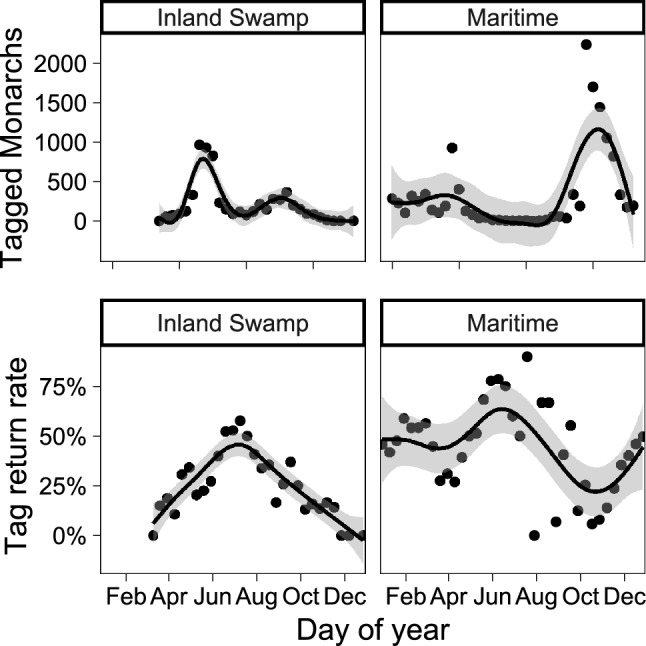
The number of tagged monarchs (top panels) and the tag return rates (bottom panels) summarized over 10-day periods across the year. Smoothed lines and grey error ribbons show results of generalized additive models (GAM) demonstrating non-linear trends. Created with ‘ggplot2’ package^44^ in R^41^.

Wild monarch butterflies were captured and tagged in all months of the year in coastal SC from 2018 to 2022, with most monarchs from maritime habitats being captured in the spring, fall, and winter, while most monarchs from inland swamp habitats were captured during the summer (Table 1; Fig. 1). Tag return rates averaged 26% across all months and habitats but varied seasonally for each habitat type (Fig. 1).

**Table 1:** Summary of monarch tagging data across habitat and season for sampling conducted from January 2018 to April 2022.

Habitat	Season	No. tagged	No. recaptured	Recapture rate
Inland swamp	Spring	2884	665	23%
Inland swamp	Summer	1785	675	38%
Inland swamp	Fall	1392	336	24%
Inland swamp	Winter	0	0	N/A
Maritime	Spring	2062	722	35%
Maritime	Summer	107	60	56%
Maritime	Fall	7621	1037	14%
Maritime	Winter	2525	1216	48%

**Figure 2:**
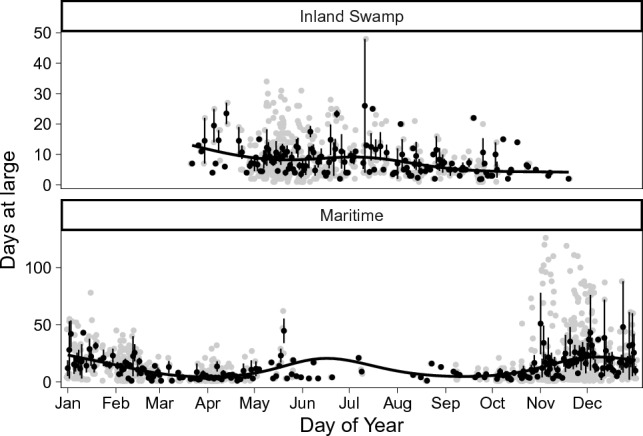
Days at large for individually tagged monarchs (grey points) and mean values (± S.E.; black points) for each ordinal day from inland swamp (top panel) and maritime (bottom panel) habitats. Smoothed lines show results of generalized additive models (GAM) demonstrating non-linear trends. Created with ‘ggplot2’ package^44^ in R^41^.

**Figure 3:**
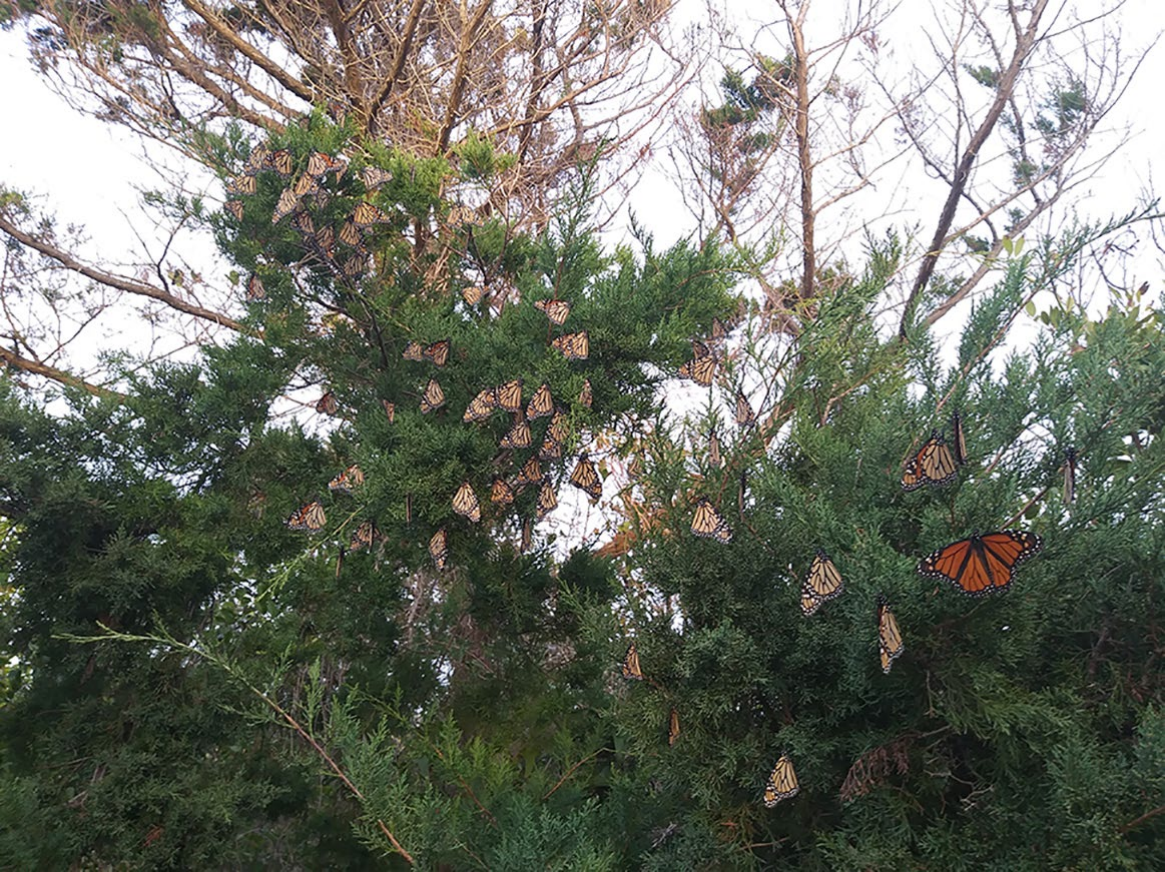
Monarch butterflies on eastern red cedar (*Juniperus virginiana*) at Folly Beach, SC during fall migration, captured on November 12, 2017. Monarchs tend to congregate on the south ends of barrier islands during the day and then migrate southward to other barrier islands with calmer winds each morning. This fall period is characterized by a high tag rate, due to the abundance of monarchs, but low tag return rates arising from migratory behavior.

**Figure 4:**
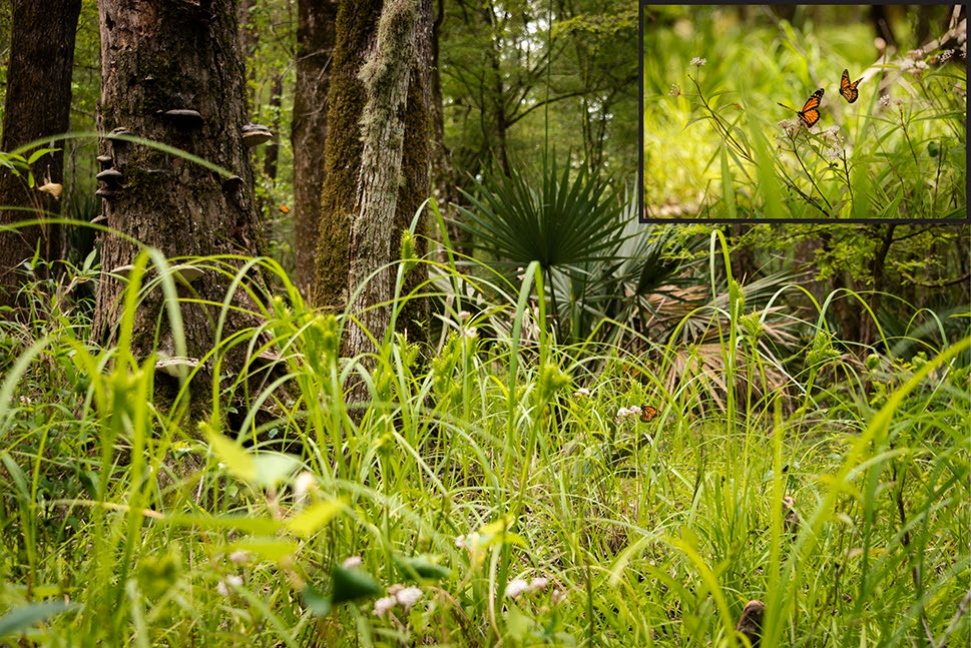
Monarch butterflies at an inland swamp in Charleston, SC captured on May 12, 2023. Monarchs are seen using aquatic milkweed (*Asclepias perennis*) and other wetland-associated plants such as bald cypress (*Taxodium distichum*), tupelo (*Nyssa* sp.), dwarf palmetto (*Sabal minor*), and sedge (*Carex* sp.). Image credit E. Weeks/SCDNR.

When comparing the number of days between capture and re-capture events (hereafter referred to as ‘days at large’, Fig. 2), we observed a significantly lower number of days at large for monarchs collected from inland swamps (6.73 ± 1.12) compared to monarchs collected from maritime areas (11.78 ± 1.95; Chi.sq = 1131.4, *P* < 0.001, SD_Year_ = 0.37). For both inland swamps and maritime habitats, days at large varied significantly by day of year (*P* < 0.001 for both habitats, Fig. 2). Monarchs were observed to congregate at the southern ends of maritime habitats (such as barrier islands) during the fall (Fig. 3) when days at large were low and southward migration was presumed to be occurring. Monarchs were observed using aquatic milkweed (*Asclepias perennis*) at inland swamp habitats (Fig. 4) throughout much of the year.

When comparing sex-specific size in spring and fall seasons across habitats, habitat, season, and their interaction were all significant factors (Table 2). Monarchs were smaller in inland swamps compared with maritime habitats for females in spring (z = − 5.11, *P* < 0.001), females in fall (z = − 11.75, *P* < 0.001; Table 3), males in spring (z = − 8.32, *P* < 0.001), and males in fall (z = − 18.25, *P* < 0.001, Table 3). Within habitats, there was no seasonal difference in monarch size between the spring and fall for females in maritime habitats (z = 0.107, *P* = 0.999), between the spring and fall for females in inland swamps (z = − 1.42, *P* = 0.489; Table 3), between the spring and fall for males in maritime habitats (z = 0.668, *P* = 0.909), nor between the spring and fall for males in inland swamps (z = − 2.20, *P* = 0.123; Table 3).

**Table 2:** Results from likelihood ratio tests of GLMM models on spatial and temporal patterns of monarch size for fall and spring seasons.

Factor	X^2^ (female/male)	*P*-value (female/male)
Location	138.15/332.87	< 0.001/< 0.001
Season	2.01/4.85	0.15/0.03
Location * season	7.07/24.12	0.008/< 0.001

**Table 3:** Estimated marginal means (± SE) from sex-specific models of monarch size variation by habitat and season.

Habitat	Season	Female (mm)	Male (mm)
Inland swamp	Fall	51.05 (0.38)	51.27 (0.28)
Inland swamp	Spring	51.82 (0.38)	52.13 (0.27)
Maritime	Fall	53.17 (0.37)	53.83 (0.26)
Maritime	Spring	53.11 (0.38)	53.59 (0.25)

### Breeding phenology and host plant phenology

Indicators of monarch breeding activity, including mating (i.e., monarchs paired at capture) and the presence of eggs, larvae, and pupae, were documented in both inland swamp and maritime habitats. Mating prevalence (i.e., the prevalence of monarchs paired at capture) during spring, summer, and fall seasons was not significantly different between inland swamps (3.3%) and maritime habitats (3.5%; z = − 0.476, *P* = 0.634). Within maritime habitats, mating prevalence in winter (0.28%) was significantly lower than fall (2.7%; z = 3.864, P < 0.001) and spring (3.1%; z = 3.639, P = 0.002), but not different from summer mating prevalence (< 0.01%; z = − 2.089, *P* = 0.157).

**Figure 5:**
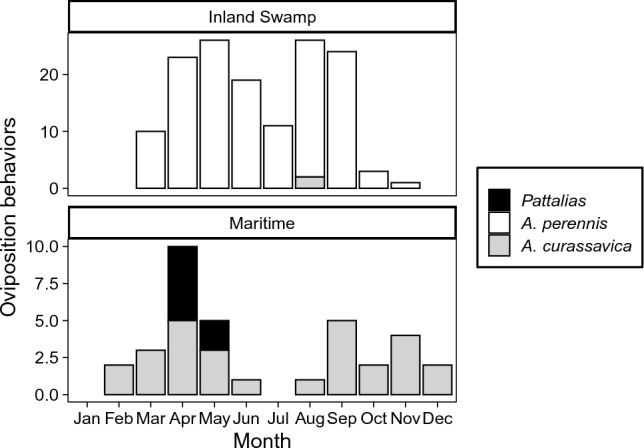
Numbers of monarchs displaying oviposition behavior in association with putative host plants (shown in black, white and grey) from maritime (top panel) and inland swamp (bottom panel) habitats by month. Created with ‘ggplot2’ package^44^ in R^41^.

**Figure 6:**
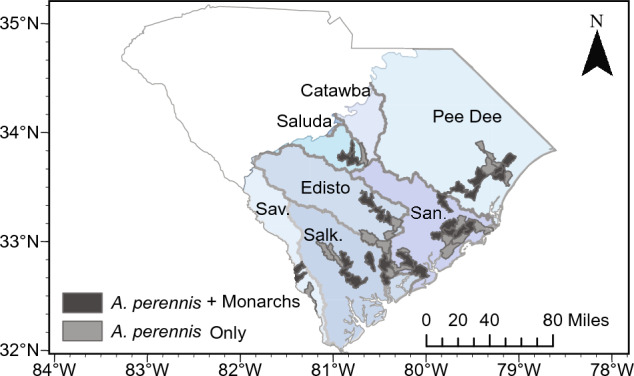
Spatial distributions and associations of aquatic milkweed (*A. perennis*) and monarchs by watershed across the coastal plain of SC created using ArcGIS Pro 3.0 (https://www.esri.com/).

Seasonal patterns of adult interactions with putative host plants (Fig. 5) were observed with swallow-wort (*Pattalias palustre* [formerly *Cynanchum angustifolium*]) representing an important plant for monarch breeding in maritime habitats in the spring. Throughout the remainder of the year, the non-native tropical milkweed, *Asclepias curassavica*, is associated with a substantial amount of monarch activity in maritime habitats. The native aquatic milkweed (*A. perennis*) was documented at inland swamps across 34 HUC-12 watersheds within the coastal plain physiographic region of SC throughout the fall, spring, and summer (Fig. 6; no captures were made in the winter when sampling was reduced). Monarch eggs, larvae, adults, and/or pupae were found in 18 of these watersheds (Fig. 6).

## Discussion

### Seasonal distribution patterns

While the population of eastern monarchs is renowned for its North American migration from Canada to overwintering grounds in Mexico^9^, an increasing body of information supports the existence of an Atlantic migratory flyway, situated east of North America’s Eastern continental divide (i.e., Appalachian Mountains). This flyway may serve as a distinct migratory route for monarchs along North America’s Atlantic seaboard^18,21–27^. The Eastern continental divide is known to represent an important barrier to dispersal for many species^28,29^, but its role as a potential barrier to monarch dispersal, such as separating putative Atlantic coast monarchs from eastern monarch populations, remains unclear^27^. The results that we present here clearly demonstrate that numerous behavioral and reproductive traits of monarchs in SC’s coastal plain differ from traits observed in eastern monarchs that overwinter in Mexico (principally those west of the Appalachian Mountains). Monarchs in the southeastern US appear to heavily use inland swamps (i.e., bottomland hardwood forests) in spring, summer, and fall months and maritime habitats in winter. These findings help highlight the range of monarch behaviors and habitat use in eastern North America.

Monarch activity and breeding during spring, summer, and fall seasons is associated with aquatic milkweed (*Asclepias perennis*), as demonstrated in the inland swamp habitats of this study. These habitats contain large expanses of aquatic milkweed that form on the floor of seasonally flooded areas of bottomland hardwood forests (*e.g.,* cypress-tupelo swamps). These systems have extreme wet-dry cycles with late fall, winter, and early spring representing periods with higher water levels when plants are likely submerged, and late spring, summer and early fall representing drier periods when plants are emergent or exposed above the water line and accessible to adult monarchs for nectaring and as a larval host plant. Aquatic milkweed is adapted to this environment by using hydrochorous (i.e., water-dispersed) seeds that can remain floating for more than 6 months, serving as an important seed bank^30^. Inland swamps also have lower minimal temperature when compared to their maritime counterparts which likely prevents monarchs from using these habitats during winter months (see below). Inland swamps are expansive throughout the southeastern US^31^. Access to these habitats for sampling, however, can be challenging, often requiring watercraft access or a willingness to wade through shallow swampy systems that have not previously been associated with heavy use by monarchs.

Our analyses indicate that aquatic milkweed and its use by monarch butterflies is extensive throughout much of the year (except winter) and is widespread across the coastal plain such that aquatic milkweed in these habitats serves as a source of recruitment for this species in the region. While aquatic milkweed has been documented as a host plant for monarchs in the past^32^, our findings show regular use of this plant throughout bottomland hardwood forests in SC. Given the expansive coverage of these habitats in the southeastern US, there is a potential for monarchs in these habitats to represent a non-negligible proportion of the eastern monarch population, but further research is needed to understand how monarch population abundances vary across this region. The extensive use of inland swamps by monarchs suggests that these habitats should be considered as part of future monarch conservation strategies.

We also highlight the role of maritime environments in the southeastern US as important overwintering habitat for monarchs. Both relatively high tag return rates and days at large for winter monarchs support this finding. Previous data have also demonstrated the use of maritime habitats in this region by overwintering monarchs^21,32^, including expansive use of the SC coast^33^. The climate of overwintering grounds in Mexico is characterized as cool and humid^34^ which, due to the buffering influence of relatively warm ocean waters on maritime habitats, generally matches the cool and humid conditions of SC’s maritime habitats. The density of overwintering monarchs in Mexico can be very high (e.g., 28 million ha^−1^^35^). And while densities of overwintering monarchs in the southeastern US are not this high, their widespread distribution in this region could represent a significant portion of the monarch population along the Atlantic seaboard. For instance, none of the monarchs tagged in this study were recovered from Mexico (although six monarchs were recaptured in Florida), suggesting that maritime habitats in the southeastern US may represent an alternative overwintering ground for monarchs. Further research is needed to quantify the population abundances, conservation status, and threats to overwintering monarchs in this region.

Swallow-wort, *Pattalias palustre*, a plant in the milkweed family Apocynaceae, is native to the coastal southeastern United States and is common in coastal grasslands with brackish soil that occur very near tidal saltmarshes^36^. This plant is also a larval host plant for the queen butterfly *Danaus gilippus*. Mating and offspring development of eastern monarchs are generally associated with milkweed plants, principally common milkweed*, Asclepias syriaca*, as well as fewflower milkweed, *Asclepias lanceolata*, swamp milkweed*, A. incarnata*, butterfly weed*, A. tuberosa*, whorled milkweed*, A. verticillata*, and poke milkweed *A. exaltata*. Monarch butterflies observed in this study showed a heavy reliance on both *A. perennis* and *P. palustre*, two members of the family Apocynaceae that grow in very distinct habitats (i.e., inland swamp and maritime habitats, respectively), but that have not previously been shown to support such extensive use by monarchs.

The introduction of the non-native tropical milkweed, *Asclepias curassavica* presents an important threat to monarchs. This plant is thought to contribute to increased reproductive activity of migrating fall monarchs^37^ and spread of disease^38^. The phenology of *Asclepias* spp. native to the southeastern US is such that *Asclepias* leaves are not generally available for monarch feeding or larval development during the winter. The non-native *A. curassavica*, however, keeps its flowers and leaves throughout much of the year, especially when provided thermal refuge from freezing temperatures^38^ as is often the case in maritime habitats. While *A. curassavica* may disrupt monarch breeding phenology^39^, eastern migratory monarchs are generally thought to be in reproductive diapause in the fall, restricting their mating activity to spring migratory and summer residency periods^18,39^. Monarch butterflies observed in this study were characterized as displaying reproductive activity during migration periods, including reproductive activity during the fall season when *A. curassavica* was not readily observable and tag recovery rates were low (i.e., an indicator of migratory behavior). Prevalence of fall mating monarchs in this study (2.7%) was consistent with previous reports of mating activity in the region (2.4%)^21^ which are also similar to spring mating levels observed in this study (3.1%). These values likely underrepresent the true mating levels since mating monarchs can be more difficult to capture because mating pairs often move to more protected areas such as higher in the canopy^21^ and suggest that fall mating by monarchs in southeastern coastal plain habitats may be more significant than previously thought.

These findings support the contention that the traits associated with Atlantic coast monarchs deserve additional attention to better understand the relationship between monarchs separated by the eastern US continental divide. How these patterns relate to the genetically distinct monarch population recognized from South Florida^26^ is currently unknown and deserves attention. In addition, quantitative surveys of monarch populations within and outside the southeastern US would provide much-needed resolution on the relative size of monarch populations. The extensive use of inland swamps (i.e., bottomland hardwood forests) and maritime habitats (i.e., barrier islands) by monarchs suggests that protections of these habitats may be critical to protecting monarchs in this region.

## Methods

### Sample collection

As part of a longer-term dataset collected by co-author JWM who has conducted capture-mark-capture of individual monarchs overwintering in maritime habitats of SC every year beginning in 1996, trait comparisons across habitats occurred from January 2018 to April 2022 using primarily aerial nets. Monarchs were marked with specialized, self-adhesive, disc-shaped, polypropylene tags with unique identifying codes and procured from Monarch Watch. Sampling occurred in and around two habitats: inland swamps and maritime habitats. Inland swamp habitats consisted of bottomland hardwood forests with cypress and cypress-tupelo swamps of the mid-Atlantic coastal plain and southern coastal plain ecoregions^40^ in northeastern Charleston County and southeastern Berkley County, as well as West Ashley, SC. Maritime habitats consisted of barrier island and sea island habitats of coastal SC and were located near open water in Folly Beach, SC, James Island, SC, and Mount Pleasant, SC in Charleston County, SC. Surveys were conducted at both habitats throughout the year, but sampling of inland swamps was diminished during winter when no monarchs were present. Seasons were defined meteorologically as follows: Winter = Dec, Jan, Feb; Spring = Mar, Apr, May; Summer = Jun, Jul, Aug; and Fall = Sep, Oct, Nov. The presence of mating behavior was documented if monarchs were paired when captured. Data are available in supplementary Table S1.

Surveys of watersheds within the coastal plain were also conducted from June 2018 to July 2022. Representative areas of inland swamps for 34 watersheds were visually surveyed during periods of low water for the presence of aquatic milkweed and any stage of monarchs (i.e., egg, larvae, pupae, and adult) and are available in supplementary Table S2. Findings are visualized by ascribing patterns based on HUC-12 watershed designations.

### Statistical approach

Generalized linear mixed effects models (GLMM) and generalized additive models (GAM) were developed in R^41^ using packages ‘lme4’^42^ and ‘mgcv’^43^, respectively, for statistical analysis of tagging, size, and oviposition data. Data are visualized using the ‘ggplot’ package^44^ in R. For the GLMM comparing days at large across habitats, we used Poisson distributions and likelihood ratio tests, as chi-squared ANOVA, to test for parameter significance using collection year as a random effect. For monarch size, we used gaussian distributions with collection month nested in year as random effects with modeled parameter values reported as estimated marginal means, which were compared across habitats in a pairwise fashion using the ‘emmeans’ package in R^45^. For the GLMM assessing patterns of mating prevalence, we used a binomial distribution using collection year as a random effect. Mating prevalence is reported from raw data and compared across habitats and seasons in pairwise fashion using the ‘emmeans’ package.

## Supplementary Information

Supplementary Table S1.

Supplementary Table S2.

## Data Availability

The datasets analyzed during the current study are available as supplementary material.
